# White Matter Survival within and around the Hematoma: Quantification by MRI in Patients with Intracerebral Hemorrhage

**DOI:** 10.3390/biom11060910

**Published:** 2021-06-18

**Authors:** Nemanja Novakovic, Joseph R. Linzey, Thomas L. Chenevert, Joseph J. Gemmete, Jonathan P. Troost, Guohua Xi, Richard F. Keep, Aditya S. Pandey, Neeraj Chaudhary

**Affiliations:** 1 Department of Neurosurgery, University of Michigan, Ann Arbor, MI 48109, USA; novakovicn3@gmail.com (N.N.); jlinzey@med.umich.edu (J.R.L.); gemmete@med.umich.edu (J.J.G.); guohuaxi@med.umich.edu (G.X.); rkeep@med.umich.edu (R.F.K.); adityap@med.umich.edu (A.S.P.); 2 Department of Radiology, University of Michigan, Ann Arbor, MI 48109, USA; tlchenev@med.umich.edu; 3 Michigan Institute for Clinical and Health Research, University of Michigan, Ann Arbor, MI 48109, USA; troostj@umich.edu

**Keywords:** intracerebral hemorrhage, magnetic resonance imaging, white matter injury, brain edema, fractional anisotropy maps

## Abstract

White matter (WM) injury and survival after intracerebral hemorrhage (ICH) has received insufficient attention. WM disruption surrounding the hematoma has been documented in animal models with histology, but rarely in human ICH with noninvasive means, like magnetic resonance imaging (MRI). A few human MRI studies have investigated changes in long WM tracts after ICH remote from the hematoma, like the corticospinal tract, but have not attempted to obtain an unbiased quantification of WM changes within and around the hematoma over time. This study attempts such quantification from 3 to 30 days post ictus. Thirteen patients with mild to moderate ICH underwent diffusion tensor imaging (DTI) MRI at 3, 14, and 30 days. Fractional anisotropy (FA) maps were used to calculate the volume of tissue with FA > 0.5, both within the hematoma (lesion) and in the perilesional tissue. At day 3, the percentages of both lesional and perilesional tissue with an FA > 0.5 were significantly less than contralateral, unaffected, anatomically identical tissue. This perilesional contralateral difference persisted at day 14, but there was no significant difference at day 30. The loss of perilesional tissue with FA > 0.5 increased with increasing hematoma size at day 3 and day 14. All patients had some tissue within the lesion with FA > 0.5 at all time points. This did not decrease with duration after ictus, suggesting the persistence of white matter within the hematoma/lesion. These results outline an approach to quantify WM injury, both within and surrounding the hematoma, after mild to moderate ICH using DTI MRI. This may be important for monitoring treatment strategies, such as hematoma evacuation, and assessing efficacy noninvasively.

## 1. Introduction

Neural functional compromise post intracerebral hemorrhage (ICH) is devastating, as it leads to a nearly 40% 1 month mortality [1,2]. ICH can cause mechanical disruption to white matter (WM), plus secondary injury inflicted by red blood cell lysis and hemoglobin degradation products [3,4]. Although animal ICH models have implicated iron as a major mediator of neurotoxicity, specific pathways of injury to WM have not been fully elucidated [3,5].

Recently, diffusion tensor imaging (DTI) has been used to examine ICH-induced injury to large WM fiber tracts, e.g., the corticospinal tract (CST) [6]. The application of DTI in ICH-related injury to WM fibers has, as yet, focused on assessing the correlation of long-term functional outcome with initial insult. Some animal ICH models have also applied DTI to assess changes in CST following minimally invasive surgery (MIS) [7]. MIS has not shown significant benefit in the human subjects with ICH in a recent randomized controlled study [8].

A recent study in a porcine ICH model, conducted by the authors, demonstrated survival of white matter fibers within the hematoma [9]. No other study to date has evaluated the extent of WM injury within the hematoma and in the surrounding tissue in human ICH. In a preliminary tractography study we conducted, WM fibers showed some crossing through the basal ganglia hematoma with steady increase to near normal symmetry with the contralateral tract over 1 month. This led us to hypothesize that (1) some WM tracts within the hematoma survive the hemorrhage, and (2) hematoma size affects the degree of initial perihematomal WM injury and the degree of recovery. Hence, to assess local injury to WM within the hemorrhage and in the surrounding tissue, we performed magnetic resonance imaging (MRI) prospectively at multiple times over a period of 1 month after ictus, and developed a novel methodology to quantify WM changes utilizing existing MRI sequences employed in routine clinical practice.

## 2. Materials and Methods

### 2.1. Patients and MRI

This study was approved by our Institutional Review Board. The patients were prospectively recruited to the study beginning in 2013. The preliminary 3 patients and one control (normal human subject) formed the pilot data to substantiate two R21 proposals to NIH to study iron quantification by MRI. Subsequently, since approval for funding of the R21s in 2017 and 2018, a total of 15 patients have been recruited. Two of these patients and the first 3 patients did not have DTI sequence acquisition. Hence, thirteen patients meeting inclusion criteria were consented for participation. Eligible patients (1) were between the ages of 18 and 85 years old, (2) had acute spontaneous supratentorial hemorrhages, and (3) were medically and neurologically stable to undergo non-contrast brain MRI at days 3, 14, and 30. Patients were excluded if their hemorrhage was the result of an intracranial aneurysm, arteriovenous malformation, trauma, hemorrhagic conversion of ischemic stroke, brain tumor, brain calcification, thrombocytopenia, or coagulopathy, or if they had bilateral hemorrhages. Additionally, patients with decreased hepatic function (ALT or AST >2.5× upper limit of normal), patients planning to undergo hematoma evacuation, patients with an active pregnancy, or patients for whom treatment or life support was being withdrawn at time of enrollment were also excluded from the study.

Brain MRI exams were performed with a 3T Research system (Ingenia ver 5.7x; Philips, Amsterdam, The Netherlands), using a 32-channel head coil and standard 3D T1-weighted, T2-weighted, and fluid-attenuated inversion recovery sequences. White matter integrity was probed using 32 direction b-values 0–800 s/mm^2^, single-shot echo planar imaging (EPI) DTI for generation of fractional anisotropy (FA) maps along with other diffusion/anisotropy metrics at 1 × 1 × 2.3 mm^3^ resolution.

### 2.2. Image Analysis

**Figure 1:**
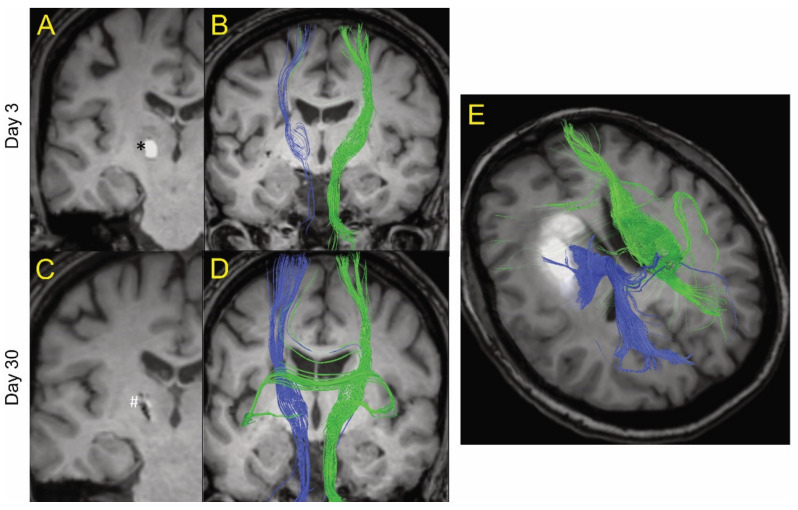
Example of MRI tractography of intracerebral hemorrhage patients. The images are postprocessed 2D axial images with projected WM fibers in 3D in a cranio–caudal direction. One patient (age 50s) is shown at day 3 (**A** and **B**) and day 30 (**C** and **D**) after ictus. Blue fibers are ipsilateral and green fibers are contralateral to the hematoma. At day 3, there was a general decrease in the fiber tracts (blue) ipsilateral to the hematoma (shown as * in **A**) compared to contralateral tracts (green). However, there was evidence of some white matter fibers crossing the hematoma. At day 30, the hematoma has largely resolved (shown as # in **C**) and there was a recovery of the ipsilateral fiber tracts. (**E**) MRI tractography of a patient (age 60s) with a larger hematoma (19.4 mL) at day 14. It too showed evidence of white matter tracts crossing the hematoma.

Images were analyzed using 3D Slicer software (available online: https://www.slicer.org/, accessed on 4 January 2021). DTI (FA, linear- and planar-anisotropy, ADC eigenvalues and trace, and b = 0, 800 diffusion-weighted imaging (DWI)) were created and stored in MetaImage (*.mhd, *.raw) format. In two patients (included in Figure 1), tractography was postprocessed on DTI images. The directionality of collective eigenvectors of the CST was assigned a color code (green), and the ipsilateral (blue) and contralateral (green) were differentiated for visual inspection. The integrity of the white matter was evaluated using FA maps. FA measurements were used indirectly to reflect changes to white matter, since it is heavily modulated by the degree of fiber unidirectionality, which varies with location in the brain. This bias was mitigated using 3D spherical segmentation volumes on FA maps to encompass the ICH lesion and the same anatomical location within the contralateral hemisphere. Voxels having an FA volume > 0.5 were used as a simplified index of directional white matter. Ill-defined voxels assigned values of non-assigned nominal (NaN) will, therefore, not interfere with data analysis. Normal and densely packed white matter fibers are completely isotropic or directional on DTI, having a value of 1. Other not so densely packed white matter fibers may display lower numbers of FA, like 0.7–0.9. To include disrupted fibers in the event of a hematoma, FA 0.5 was thought to be a good comprehensive threshold for calculations and would reflect the proportion of WM with relatively retained directionality. The bias was further mitigated by comparing ipsilateral volumes to their contralateral equivalents (difference in contralateral and ipsilateral volume > 0.5 FA). The radius of the spheres, drawn as areas of interest within the ICH and within the contralateral hemisphere, ranged between 2.0 and 2.5 cm. Sphere size was chosen with consideration to the lesion location, size, and surrounding structures, being careful not to cross the midline. Diameters of the spherical segmentation volumes were held constant for each subject to quantify evolution over time. The ipsilateral sphere was divided into lesional (the hematoma itself) and perilesional (defined as the surrounding tissue of the hematoma up to the edge of the sphere of interest) volumes, where lesional volume was defined by the boundaries of the lesion (hematoma) using DTI b = 0 labeled maps and then superimposing those labeled maps on the corresponding FA series map.

Apart from the FA volume > 0.5 (milliliters), the following parameters were calculated: tissue FA volume > 0.5 (percent), and the volume of tissue with FA > 0.5 as a percentage of the total volume of tissue. For the ipsilateral and contralateral spheres and the lesion, this parameter was measured directly. For the perilesional zone, it was determined from the difference between the ipsilateral sphere and the lesion. Further, the difference in expected and measured tissue with FA > 0.5 in the perilesional tissue was calculated as a measure of white matter loss. That parameter was: (ipsilateral sphere FA > 0.5 (milliliters) − lesion FA > 0.5 (milliliters)) − (peri-lesion volume (milliliters) × contralateral sphere FA > 0.5 (milliliters)/contralateral sphere volume (milliliters)).

### 2.3. Statistics

All statistical analyses were performed using GraphPad Prism 9 (https://www.graphpad.com, accessed on 30 May 2021). Normally distributed data are presented as mean ± standard deviation (SD). A mixed effects model was used to compare FA > 0.5 values in different regions of interest across different time points. Changes in lesional and perilesional FA > 0.5 over time were analyzed using paired *t*-tests. The relation between hematoma size and FA > 0.5 (days 3, 14, and 30) was analyzed by regression analysis. Regression analyses of the amount of FA > 0.5 tissue lost against hematoma size at different time points were compared by analysis of covariance (ANCOVA). Differences were considered significant at *p* < 0.05 (two-tailed). Variance components covariance structure was used based on smallest Akaike information criterion of null models (i.e., no fixed effects) with various covariance structures. Model results are shown in Supplementary Table S1. Model 1—which uses all observations but does not adjust for time—shows all locations were higher than the lesion, though the strongest difference was contralateral sphere vs. lesion. Results changed little in Model 2, which also adjusted for time. However, Model 3 introduces an interaction between location and time—positing if the differences between locations are stronger at certain time points. No significant interaction was observed (*p* = 0.5222). We have modified our interpretations/conclusions to be that there are differences by location improvements over time, but that the differences in location remain the same over time (at least with respect to these 3 early timepoints) (Supplementary Table S1).

## 3. Results

All recruited patients had hematomas located in the basal ganglia region, except for one patient who had a lobar hemorrhage with intraventricular hemorrhage (IVH). DTI data were acquired on day 3 in 11 subjects, day 14 in 10, and day 30 in 9. Seven patients had DTI data at all three time points (days 3, 14, and 30). Hematoma sizes ranged between 0.3 and 39 mL. The smaller hematomas had good clinical recovery. Clinical follow-up ranged from 4 months to 2 years 64 days. The larger hematomas were associated with poor outcomes, with a modified Rankin Scale (mRS) score of 4 in the patient with a 39 mL hematoma at 11 month follow-up. Another patient with a 5 mL hematoma had an mRS of 3 at 6 month follow-up. All other patients had an mRS of 0–2 at latest follow-up. Two patients with 9 and 19 mL hematomas had to be intubated at onset, but made a good recovery to an mRS of 0–2 at latest follow-up (Table 1).

Figure 1 shows MRI tractography of two ICH patients. Figure 1A–D show the MRI tractography of a patient (age 50s) with a 0.3 mL hematoma at days 3 and 30 post ictus. At day 3, visually, there was evidence of a reduction in white matter tract fiber density ipsilateral to the hematoma compared to contralateral. Interestingly, some of the visualized ipsilateral WM tracts actually traverse the ICH. By day 30, there was near complete resolution of the hematoma and an apparent recovery of white matter tract fiber density ipsilateral to the lesion as grossly visualized. Figure 1E shows the MRI tractography of a patient (age 60s) with a larger hematoma (19.4 mL) at day 14. It too shows evidence of white matter tracts crossing the hematoma. These results led us to try to quantify the presence of organized white matter (here defined as FA > 0.5) both within the hematoma and around the lesion over time after ictus. The primary goal of the analysis was to analyze the percentage change between two time points for lesion and peri-lesion volume. The authors hypothesize that, in a larger sample of patients, it will be useful to analyze the above data adjusted for covariates like age, gender, and hematoma size; however, given the small sample in our study, this function was thought not to be scientifically meaningful.

**Figure 2:**
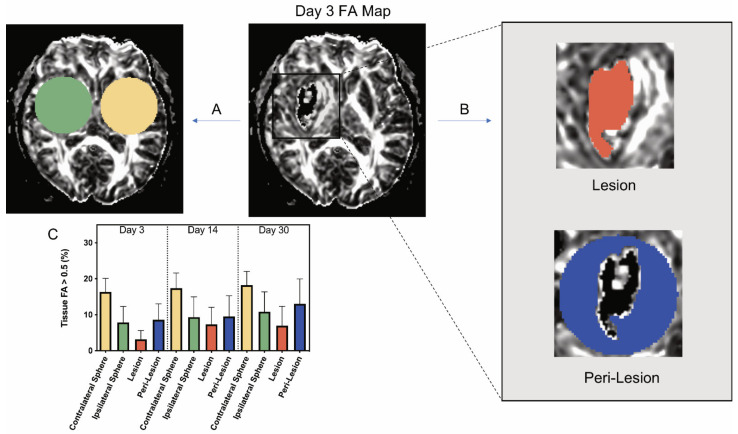
Example of analysis of a day 3 fractional anisotropy (FA) map and changes in the amount of tissue with FA > 0.5 ipsi- and contralateral to the intracerebral hemorrhage in patients at different times. (**A**) On an FA map, a sphere centered on the lesion (green) was analyzed, along with an identical contralateral sphere (yellow). (**B**) The ipsilateral sphere is split into two components, the lesion volume and peri-lesion volume. The lesion volume FA > 0.5 was directly measured, and the peri-lesion volume was calculated using the difference between the ipsilateral sphere and lesion data (see Methods). All FA > 0.5 data were expressed as a percentage of tissue volume. (**C**) Graph showing the percentage of tissue FA > 0.5 for all of these areas of interest at days 3, 14, and 30. Values are means ± SD; *n* = 9–11. Using a mixed effects model, significant differences were seen by location. All pairwise comparisons for location were significant with *p* < 0.0001 (Bonferroni adjusted threshold for 6 comparisons = 0.0083) with the exception of ipsilateral sphere vs. peri-lesion (*p* = 0.26). There were also improvements over time (*p* = 0.0053), but no interaction between location and time (*p* = 0.52). (See Supplemental Table S1 for full details).

Thirteen patients received MRI scans at days 3, 14, or 30. The demographics of the patients are shown in Table 1. Although initial plans included scans at each time point, some patients did not receive each scan. The volume of tissue with FA > 0.5 in a contralateral and an ipsilateral sphere was determined at each time point, with the latter centered on the hematoma (Figure 2A). The ipsilateral sphere was then divided into the lesional and perilesional volumes (Figure 2B). The volume with FA > 0.5 was determined within the lesion, while the perilesional value was calculated from the difference between the ipsilateral sphere and the lesion (see Methods).

At all three time points, the percentage of the contralateral sphere volume occupied by tissue with FA > 0.5 was similar across all study patients (~17%; Figure 2C) and greater than the FA > 0.5 volume within the ipsilateral sphere. The percentage of perilesional volume with FA > 0.5 at both days 3 and 14 was significantly less than the percentage of contralateral volume with FA > 0.5: day 3, 8.61 ± 4.42% vs. 16.33 ± 3.77% and day 14, 9.53 ± 5.76% vs. 17.39 ± 4.22% (*p* < 0.001; Figure 2C). However, comparisons of volume with FA > 0.5 in the perilesional region versus the same sized contralateral regions did not vary significantly at day 30 post ICH. All the ICH volumes examined in this study had some lesional volume with FA > 0.5 at all time periods. At day 3, the ICH volume with FA > 0.5 was significantly less than the perilesional volume with FA > 0.5 (3.15 ± 2.47% vs. 8.61 ± 4.42%, *p* < 0.05; Figure 2C). At days 14 and 30, there was no significant difference between the percentage of lesional volume and the percentage of perilesional volume associated with FA > 0.5 (Figure 2C).

**Figure 3:**
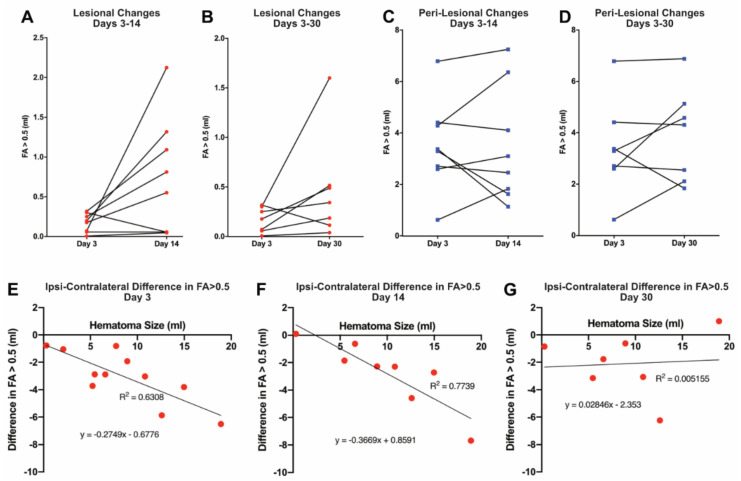
(**Upper panel**) Temporal profile of intracerebral hemorrhage-induced injury in patients who received multiple scans. The graphs show changes in the volume (milliliters) of tissue FA > 0.5 within the lesion between days 3 and 14 (**A**) and days 3 and 30 (**B**), and changes in the volume (milliliters) of tissue FA > 0.5 for the peri-lesion between days 3 and 14 (**C**) and days 3 and 30 (**D**). There was no statistical significance between the volume of tissue FA between any of these days (**A**: *p* = 0.061, **B**: *p* = 0.153, **C**: *p* = 0.961, **D**: *p* = 0.351; paired *t*-tests, two-tailed). (**Lower panel**) Effect of hematoma size on intracerebral hemorrhage-induced white matter injury. Contralateral FA > 0.5 tissue was used to obtain the expected amount (milliliters) of perilesional FA > 0.5 tissue and, from the measured amount of perilesional FA > 0.5 tissue, to calculate the amount (milliliters) lost (see Methods). Perilesional tissue loss is shown at days 3, 14, and 30 (**E**, **F**, and **G**, respectively) plotted against hematoma volume (milliliters) at day 3. The amount of FA > 0.5 tissue loss was significantly correlated with hematoma size at days 3 (*p* < 0.01) and 14 (*p* < 0.01), but not at day 30 (*p* = 0.878). Two patient data points are absent from graphs **F** and **G**, as these patients did not have the initial hematoma size at day 3 available for reference.

To examine the temporal profile of the ICH-induced injury, changes in the proportion of lesional and perilesional tissue with FA > 0.5 were calculated in those patients who received multiple scans (Figure 3). Within the lesion, there was no evidence of a generalized decrease in the amount of tissue with FA > 0.5 between days 3 and 14 (Figure 3A), or between days 3 and 30 (Figure 3B). Indeed, only one of eight patients actually showed a decline in the proportion of lesional volume with FA > 0.5 at day 14 when compared to day 3, and one of seven patients with imaging at 30 days had a decline in FA > 0.5 lesional volume when compared to day 14 volumes. Similarly, quantitatively, there tended to be an increase at both times, although this did not reach significance (*p* = 0.061 and *p* = 0.153, respectively). In the perilesional area, there was marked heterogeneity in the amount of tissue with FA > 0.5, with four of eight patients showing a decrease by day 14, and three of seven by day 30; however, some patients showed marked increases (Figure 3C,D), such as the patient shown in Figure 1. Understanding the mechanisms underlying that heterogeneity will be very important.

The impact of initial hematoma size (determined at day 3) on the evolution of the ICH-induced injury was examined. Using the contralateral FA > 0.5 to calculate the expected amount of perilesional tissue with FA > 0.5 allowed the determination of the amount of such tissue lost after ICH (milliliters). By regression analysis, the amount of FA > 0.5 tissue lost increased significantly with hematoma size at days 3 and 14 (*p* < 0.01 at both days; Figure 3E,F). The two regressions were not significantly different by ANCOVA, suggesting that the loss of perihematomal tissue with FA > 0.5 was maximal by day 3. However, there was no significant correlation at day 30 (Figure 3G). The latter may reflect the recovery of white matter tracts in some patients (e.g., Figure 1).

With respect to the lesion itself, the percentage of the hematoma with FA > 0.5 did not vary with hematoma size at day 3. At that time, the mean FA > 0.5 (percent) was 3.15 ± 2.47%, and FA > 0.5 (percent) = −0.19 × (hematoma volume) + 4.75 (R^2^ = 0.179; *n* = 11).

## 4. Discussion

This study outlines a methodology to quantify perilesional white matter survival/injury and the persistence of apparent WM within the hematoma after ICH, using serial MRI with DTI 3–30 days post ictus. The study shows that all but two patients had an mRS score of 0–2 at latest follow-up after ICH. Moreover, in ICH patients, apparent WM fibers (FA > 0.5) persisted within the lesion for at least 30 days. In addition, initial perilesional WM loss correlated with hematoma size, but there was considerable variation in how that changed with time, with some patients showing considerable recovery.

Although clinical outcome should not be given too much weight in such a small cohort, the case mix shows that some patients with medium-sized hematomas (15–20 mL) can have varied presentation, even with intubation, but still have good recovery. Neuronal function, specifically recovery from hemiplegia, can be varied (Table 1). This variation raises the possibility of different mechanisms at play dictating initial neuronal functional loss and subsequent recovery for similar sized hematomas. This clinical nuance has not been critically evaluated to date, as there are no existing imaging or other biomarkers to assess for and predict functional recovery. The authors believe that the demonstrated MRI-based assessment of neurotoxicity mediated by iron overload and extent of WM tract injury may inform assessment of functional loss, and that its tracking over a period of 1 month could provide a prognostic indicator.

DTI is an advanced form of diffusion-weighted imaging of the brain that assesses the integrity of white matter fibers [6]. There are several publications tracking recovery of motor function in acute ischemic stroke with DTI. Although the exact mechanism causing damage to the white matter tracts in the ischemic tissue bed is different from that in the hemorrhagic bed, motor function recovery following ischemic stroke has been correlated with improvement in FA values on DTI [10,11]. As has been demonstrated in ischemic stroke, DTI imaging holds promise for an improved understanding of the natural history of hemorrhagic stroke. In non-WM brain tissue, the protons and collective eigenvectors are isotropic (FA = 0) and, hence, are not directional on DTI MRI. In contrast, intact WM fibers with good directionality are entirely anisotropic (FA = 1). Hence, for the purposes of our analysis, it was deemed that relatively healthy WM with integrity and good directionality would have an FA above a 0.5 threshold. Using this threshold, WM was detected within the hematomas of all patients at 3 days, and it persisted to at least 30 days. There is a possibility that some structure other than WM might result in an FA > 0.5 within the hematoma. However, an examination of FA during hematoma formation in vitro reported FA values of 0.1–0.2 from day 1 to day 56 [12], suggesting this is unlikely. Histology on human ICH would help to confirm the presence of such intra-hematomal WM.

Due to mass effect, some hematomas are evacuated in routine clinical practice. An open or minimally invasive surgical evacuation is performed assuming no residual healthy tissue within the hematoma. Our study suggests the presence of residual WM within the hematoma at day 3 that is potentially at risk from injury during surgical intervention. The risk to such tissue may depend on the type of surgical evacuation.

Perilesional WM fibers are disrupted and deranged following an ICH. In a recent study on mini pigs, Yang et al. [13] demonstrated WM derangement on the ipsilateral hematoma side compared to the contralateral hemisphere. They correlated the FA values to axonal number counts on histology. Tao et al. [14] looked at day 4 MRI with DTI in 32 patients with supratentorial ICH, examining CST FA and comparing to the contralateral normal side at five slices below the level of the ICH. They found the clinical ICH score to be more predictive of functional outcome than the relative FA values on DTI [14]. They found no difference in mean FA between ipsi- and contralateral CST at day 4. They accounted for the lack of difference to heterogeneity of the population and recommended studies with larger patient numbers and multiple time-point assessment of DTI parameters. Other studies demonstrate decreased ipsilateral FA within 5 days of ICH onset [15,16,17,18]. Goh et al. [19] demonstrated the value of depicting perilesional white matter integrated with anatomical MRI and proposed its utility in assessing WM damage following ICH. The method of FA assessment in our study is unique and better normalized to the contralateral side and has multiple time points over a period of 1 month. In addition, our study is the first of its kind to demonstrate relatively healthy WM fibers within the hematoma.

Our study found that the percentage of perilesional tissue with FA > 0.5 was significantly reduced compared to contralateral at days 3 and 14. The amount of such tissue lost at those times correlated with initial hematoma volume (Figure 3). At day 30, there was no such correlation and the percentage of perilesional tissue with FA > 0.5 was not significantly reduced compared to contralateral. This may reflect an apparent recovery in WM with time that was noted in some but not all patients (Figure 1 and Figure 3). The cause of this variability is unknown. It might possibly be explained by different degrees of iron leaching from both within and around the hematoma. The authors propose to study this aspect in more detail to evaluate the links between the quantified iron by MRI [20] and the FA > 0.5 tissue at different times following hematoma onset. We speculate that, when studied in a large number of human subjects, there may be a correlation between MRI-calculated iron levels and the extent of WM injury and recovery after ICH. A wider detailed DTI analysis with whole-brain segmentation could be undertaken to better evaluate changes in WM tracts remote from the site of hematoma over 1 month following ICH. The authors believe that it can be well studied in a larger cohort of patients and potentially can be correlated with cognitive evaluation in a future study. In our cohort, there was only one patient with an IVH. The authors hypothesize that the pattern of WM disruption in the periventricular region can be bilateral in IVH, and also needs detailed analysis in a dedicated large cohort of patients.

This study has several limitations, including the small number of patients and variations in MRI acquisition (missing time points). However, the authors believe the observations are significant for future study planning. Such studies would include a large number of patients with multiple MRI time points to better evaluate the relationship of tissue iron released from the hematoma and the specific derangement in WM fibers.

## 5. Conclusions

MRI-based DTI image acquisition provides a noninvasive quantifiable method of evaluating WM within the hematoma and surrounding tissue in mild to moderate ICH. Some white matter appears to persist within the hematoma after mild to moderate ICH for at least 1 month. This should be taken into account in clot evacuation strategies. Our observations and methodology will also inform future studies with larger patient numbers designed to assess, noninvasively, the effects of treatment strategies on perilesional WM injury after mild to moderate ICH.

## Figures and Tables

**Table 1:** Demographics and neurological exam results.

Patient	Age (Years)/Gender	Hematoma Size, T2* (mL) (Day)	Onset Neurological Exam	Latest Neuro Exam (Time Since Onset)	Point Change in Neuro Exam	Latest mRS
01	60s/F	39.036 (14)	Left facial droop, left hemiparesis, LUE and LLE 0/5, slurred speech, NIHSS 17	LUE and LLE 2/5 (11 months)	UE: +2, LE: +2	4
02	50s/M	2.354 (14)	Diff. using left hand and slurred speech, LUE and LLE 4+/5	Full strength 5/5 (2 years, 64 days)	UE: +1 (full rec.), LE: +1 (full rec.)	1
03	40s/F	8.932 (3)	Intubated, right side only localizes, RUE 0/5, withdraws with RLE 0/5	RLE and RUE 5/5 (16 months)	UE: +5 (full rec.), LE: +5 (full rec.)	1
04	50s/M	0.308 (3)	Left facial asymmetry	Facial palsy persisted (4 months)	NA	1
05	60s/M	5.2325 (3)	NIHSS 8, left hemiparesis and slurred speech, LUE 0/5, LLE 1/5	LUE 1/5 and LL 5/5 (6 months)	UE: +1, LE: +4 (full rec.)	3
07	60s/F	11.4862 (1)	Expressive aphasia, RLE 4/5 and R pron drift, mRS 4	5/5 (8 months)	LE: +1 (full rec.)	0
08	20s/F	18.9037 (3)	Right-sided hemiplegia, intubated, RUE and RLE 0/5	RUE 4/5 and RLE 5/5 (56 days)	UE: +4, LE: +5 (full rec.)	2
09	30s/F	5.4602 (3)	Left facial droop and slurred speech, left hand and arm weak, LUE 4/5, LLE 5/5	LUE and LLE 5/5 (60 days)	UE: +1 (full rec.), LE: No change	0
10	70s/F	2.1022 (3)	Seizure at presentation, headache, mRS 0, aphasia, no weakness	5/5 (75 days)	UE: no change, LE: no change	0
11	80s/F	6.578 (3)	Left facial droop and dysarthria, NIHSS 3, mRS 0-2, LUE 4/5, LLE 5/5	LUE 5/5, LLE 4/5 (120 days)	UE: +1 (full rec.), LE: −1	2
12	60s/M	14.973 (3)	mRS 0, 5/5	5/5 - L facial droop persists (4 months)	UE: no change, LE: no change	1
13	60s/F	10.8767 (1), 10.8146 (3)	Left-sided hemiparesis, facial droop, NIHSS 11, mRS 3, LUE 3/5, LLE 4/5	LUE and LLE 5/5 (2 months)	UE: +2 (full rec.), LE: +1 (full rec.)	1
15	60s/M	13.7747 (1), 12.6063 (3)	Left-sided hemiplegia, trace withdrawal LUE, triple flex LLE	LUE 0/5, LLE 2/5, mRS 4	UE: no change, LE: +2	4

Abbreviations: LE, lower extremity; LLE, left lower extremity; LUE, left upper extremity; mRS, modified Rankin Scale score; NIHSS, NIH Stroke Scale; RLE, right lower extremity; RUE, right upper extremity; UE, upper extremity. T2* is a gradient echo MRI sequence which was used to measure hematoma volume.

## Data Availability

Data are available upon reasonable request from the corresponding author.

## Supplementary Materials

The following are available online at https://www.mdpi.com/article/10.3390/biom11060910/s1, Table S1: Linear mixed-effects model results.
