# Supplementary material for: White Matter Survival within and around the Hematoma: Quantification by MRI in Patients with Intracerebral Hemorrhage
Source: Biomolecules. 2021 Jun 18;11(6):910. doi: 10.3390/biom11060910 (PMC8234588; doi:10.3390/biom11060910)
Supplement: Supplementary file 1 [file biomolecules-11-00910-s001.zip › biomolecules-1230370-supplementary.pdf]

Supplementary Table S1. Linear mixed-effects model results.

Variable	Model 1: Location		Model 2: Location + Time		Model 3: Location * Time Interaction	
	B [95% CI]	p	B [95% CI]	p	B [95% CI]	p
Intercept	5.74	---	4.56	---	3.65	---
Location*		<0.0001		<0.0001		<0.0001
Contralateral sphere vs. lesion	11.57 [9.76, 13.38]	<0.0001	11.57 [9.83, 13.31]	<0.0001	13.18 [10.29, 16.07]	<0.0001
Ipsilateral sphere vs. lesion	3.56 [1.75, 5.36]	0.0002	3.56 [1.81, 5.30]	0.0001	4.70 [1.81, 7.59]	0.0017
Peri-lesion vs. lesion	4.55 [2.75, 6.36]	<0.0001	4.55 [2.81, 6.30]	<0.0001	5.46 [2.56, 8.35]	0.0003
Day		---		0.0053		0.0056
14 vs. 3	---	---	1.43 [-0.18, 3.04]	0.0806	3.70 [0.66, 6.73]	0.0174
30 vs. 3	---	---	2.81 [1.13, 4.48]	0.0012	3.33 [0.20, 6.46]	0.0372
Location*day (interaction)		---		---		0.5222
Contralateral sphere vs. lesion * day 14	---	---	---	---	-3.11 [-7.30, 1.09]	0.1446
Contralateral sphere vs. lesion * day 30	---	---	---	---	-1.91 [-6.22, 2.41]	0.3824
Ipsilateral sphere vs. lesion * day 14	---	---	---	---	-2.71 [-6.90, 1.48]	0.2030
Ipsilateral sphere vs. lesion * day 30	---	---	---	---	-0.81 [-5.12, 3.51]	0.7116
Peri-lesion vs. lesion * day 14	---	---	---	---	-3.25 [-7.44, 0.94]	0.1271
Peri-lesion vs. lesion * day 30	---	---	---	---	0.60 [-3.71, 4.91]	0.7822
Random effects (variance components structure)						
Subject	11.38 [5.50, 35.93]	---	10.42 [5.04, 32.83]	---	10.41 [5.03, 32.86]	---
Residual	12.47 [9.68, 16.68]	---	11.58 [8.97, 15.54]	---	11.67 [8.97, 15.81]	---

*All pairwise comparisons for location were significant with $p < 0.0001$ (Bonferroni adjusted threshold for 6 comparisons = 0.008) with the exception of ipsilateral sphere vs. peri-lesion ($p = 0.26$).
